# Multi-gate neuron-like transistors based on ensembles of aligned nanowires on flexible substrates

**DOI:** 10.1186/s40580-024-00472-z

**Published:** 2025-01-18

**Authors:** João Neto, Abhishek Singh Dahiya, Ravinder Dahiya

**Affiliations:** 1 James Watt School of Engineering, University of Glasgow, Glasgow, G12 8QQ UK; 2 Bendable Electronics and Sustainable Technologies (BEST) Group, Electrical and Computer Engineering Department, Northeastern University, Boston, MA 02115 USA

**Keywords:** Multi-gate transistors, Dielectrophoresis, Flexible electronics, Neuromorphic, Electronic skin

## Abstract

The intriguing way the receptors in biological skin encode the tactile data has inspired the development of electronic skins (e-skin) with brain-inspired or neuromorphic computing. Starting with local (near sensor) data processing, there is an inherent mechanism in play that helps to scale down the data. This is particularly attractive when one considers the huge data produced by large number of sensors expected in a large area e-skin such as the whole-body skin of a robot. This underlines the need for biological skin like processing in the e-skin. Herein, we present multi-gate field-effect transistors (*v*-FET) having capacitively coupled floating gate (FG) to mimic some of the neural functions. The *v*-FETs are obtained by deterministic assembly of ZnO nanowires on a flexible substrate using contactless dielectrophoresis method, followed metallization using conventional microfabrication steps. The spatial summation of two presynaptic inputs (applied at multiple control gates) of the transistor confirm their neuron-like response. The temporal summation (such as paired-pulse facilitation) by presented *v*-FETs further confirm their neuron-like mimicking with one presynaptic input. The temporal and spatial summation functions, demonstrated by the *v*-FET presented here, could open interesting new avenues for development of neuromorphic electronic skin (*v*-skin) with possibility of biological-skin like distributed computing.

**Supplementary Information:**

The online version contains supplementary material available at 10.1186/s40580-024-00472-z.

## Graphical Abstract

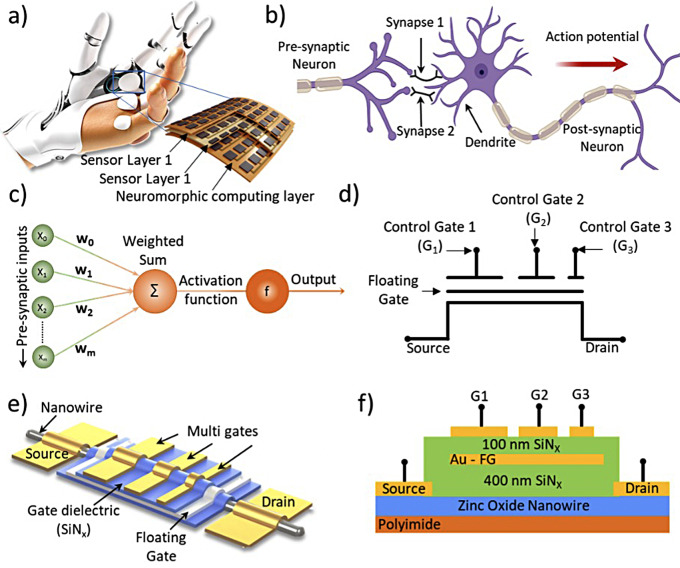

## Introduction

**Fig. 1:**
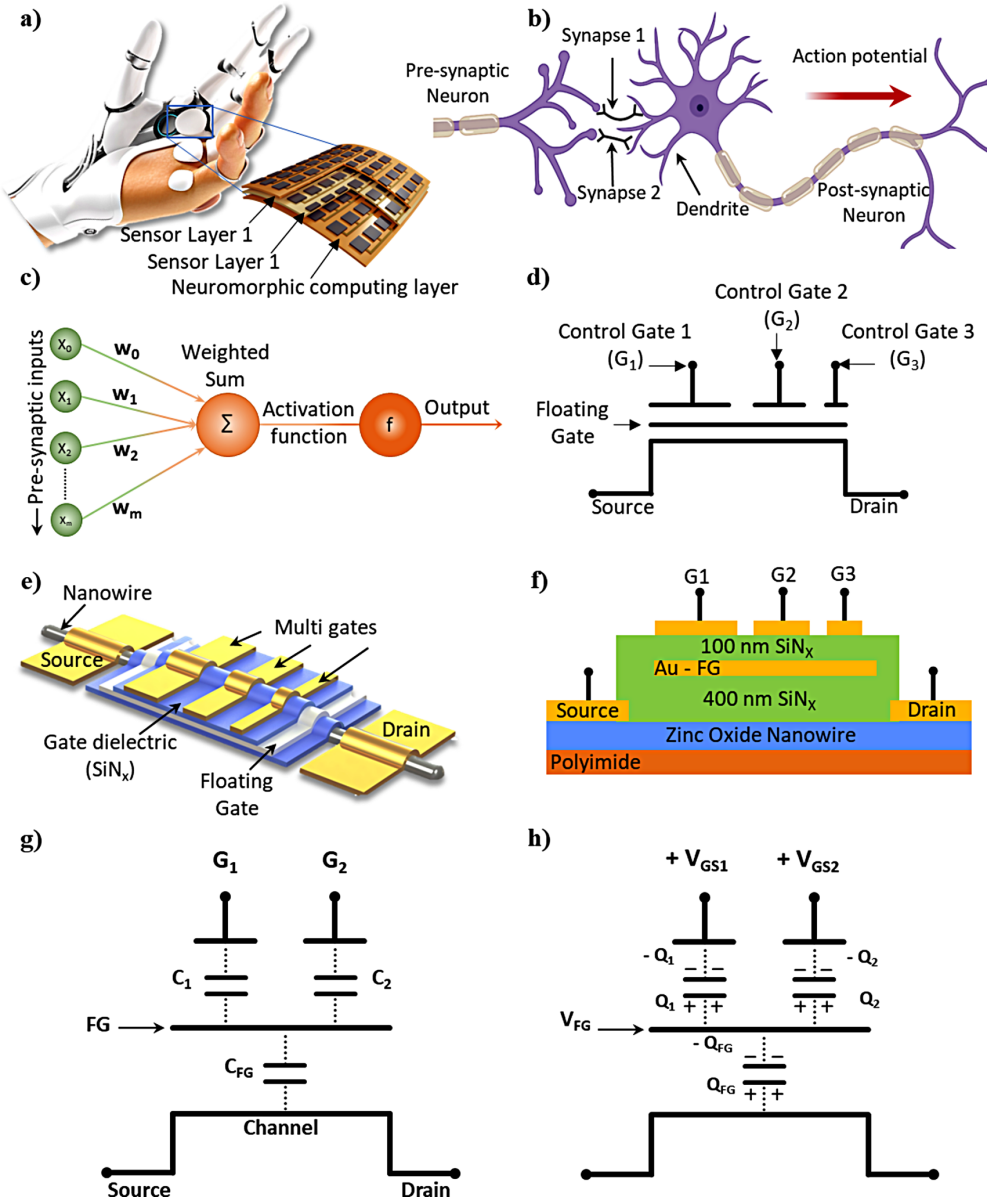
Comparison of biological neural system and the neuron like electronics: (**a**) neuromorphic electronic skin, and (**b**) schematic diagrams of the biological neurons showing synapses. The transmission of action potentials via synapse is the basis of most communication in the nervous system. (**c**) Schematic diagrams of the artificial neuron like processing. (**d**) symbol of multi-terminal transistor with a floating gate. (**e**) schematic diagram of the nanowire (NW) based multi-gate field effect transistor (FET) with a floating gate electrode to perform summation function. (**f**) Cross-section view of the multi-gate NWFET. Working mechanism of the multi-gate *v*-FET under (**g**) no control gate bias, and (**h**) control gate bias conditions

Distributed sensor-laden electronic-skin (e-skin) type interfaces are needed in a wide range of applications including human health monitoring, prosthetics, robotics, smart homes, and others [1–8]. While performing complicated tasks (e.g., object handling by robots, tactile image reconstruction), e-skin generates large datasets, which need to be handled energy efficiently and often in real time [1, 2, 9–13]. Similar issues faced in vision and auditory sensing led to emulating the spiking neural and neuromorphic mechanisms [14] and similar approaches for tactile perception have started to receive attention, as reflected from various algorithms, explored recently, for processing of tactile information such as support vector machine (SVM), linear discriminant analysis (LDA), k-nearest neighbors (kNN), spiking neural network (SNN), Extreme Learning Machine (ELM), and Bayesian analysis [15, 16]. However, due to the lack of large-scale parallel processing, the software-programmed neural networks are slower and energy-inefficient [17]. In this framework, brain-inspired or ‘Neural-like Architecture’ implemented in hardware offers considerable potential. Besides overcoming the inherent energy and speed limitations of conventional von Neumann based computing architecture, the neural-like hardware could make it possible to locally (i.e., near to the sensor) process the data [18–21]. As a result, neuromorphic computing is attracting considerable attention. The hardware implementations of neuromorphic computational building blocks such as transistors, memory devices etc. in a flexible form factor could provide a practical solution for processing of large data-set generated by various types of physical, chemical or biological sensors used in systems such as e-skin for robotics and health monitoring patches [9, 22]. Such a neuromorphic e-skin (*v*-skin), as shown in Fig. 1a, can potentially offer human skin–like and/or peripheral nervous system–like perceptual functionalities.

The hardware implementations of neuromorphic computation blocks include devices such as two-terminal memristors and three-terminal transistors on soft and bendable substrates [9, 23, 24]. One of the critical neuron functionalities is to perform summation and integration of multiple presynaptic inputs. The summation of inputs (both spatial and temporal) polarizes the soma until it reaches a threshold value where the action potential is triggered (Fig. 1b). A typical mathematical model of neuronal dendritic integration is depicted in Fig. 1c, where multiple inputs are weighted and summed up to provide an action potential by means of a mathematical function. A multi-terminal device such as transistor (Fig. 1d) could mimic similar behaviour and could possibly synchronize multimodal sensory information (e.g., touch, vision, and sound etc.) too [25]. With respect to two terminal devices such as memristors, these devices have advantages such as high stability, efficient computation, controllable testing parameters, and clear operation mechanism. Further, they can be constructed using a wide variety of materials and fabrication approaches including direct-ink writing, lithography-based microfabrication etc. on flexible substrates [8, 26]. Accordingly, multi-gate neuronal transistors based on nanoscale materials (e.g., quasi 1D semiconductors [23], and 2D transition metal dichalcogenides (TMDCs) [27] etc.) have been developed with device structures such as electrolyte-gated transistor [28, 29], ferroelectric transistor [30], and floating-gate field effect transistor (FG-FET) [31]. Among them, the FG-FETs, which is conventionally used as non-volatile memory devices in solid-state drives, show greater potential to act as a neuron-like transistor [32–34].

Motivated by this background, herein, we report ZnO nanowires-based neuron-like field-effect transistors (*v*-FET) with multiple control gates (CG) that are capacitively coupled to a floating gate (Fig. 1e, f). The structure of the *v*-FET is inspired from a neuron metal oxide semiconductor field effect transistors (MOSFET) devices reported previously [35]. In a traditional floating gate memory cell, the data is stored by trapping the electrons on floating gate (FG), which tunnel through the oxide layer onto the FG. This charge remains even when power is removed, making it a non-volatile memory-type device, like flash memory. In the present *v*-FET, however, multiple input control gates are interacting with the FG *via* different capacitive couplings and the potential of the FG controls the formation of the channel underneath the gate oxide. Thus, in our case it is assumed that no charge injection occurs through tunnelling during the device operation.

## Results and discussions

### Fabrication and working principle of a multi-gate *v*-FET

The NW *v*-FETs are designed and fabricated to imitate working of the biological neurons in a simplified manner. In this device configuration, the FG is capacitively coupled to several top CGs, and the summed electric potential at the FG modulates the channel current. The overlap width of the individual top CGs over the FG defines their respective capacitances (Fig. 1g), thereby predetermining the initial synaptic weight of the neural input [23, 36]. In an analogy with the biological neuron, the top CGs can be seen as the pre-synaptic membranes where the applied gate voltages (V_GS_) are defined as pre-synaptic inputs. Whereas the drain-source current (I_DS_) can be seen as the post-synaptic current (PSC), which is controlled by the electrical charges stimulated by the applied signals on the gate terminal (Fig. 1h). This simulates how neurons process incoming signals and transmit information to downstream neurons when the appropriate conditions are met [37, 38].

**Fig. 2:**
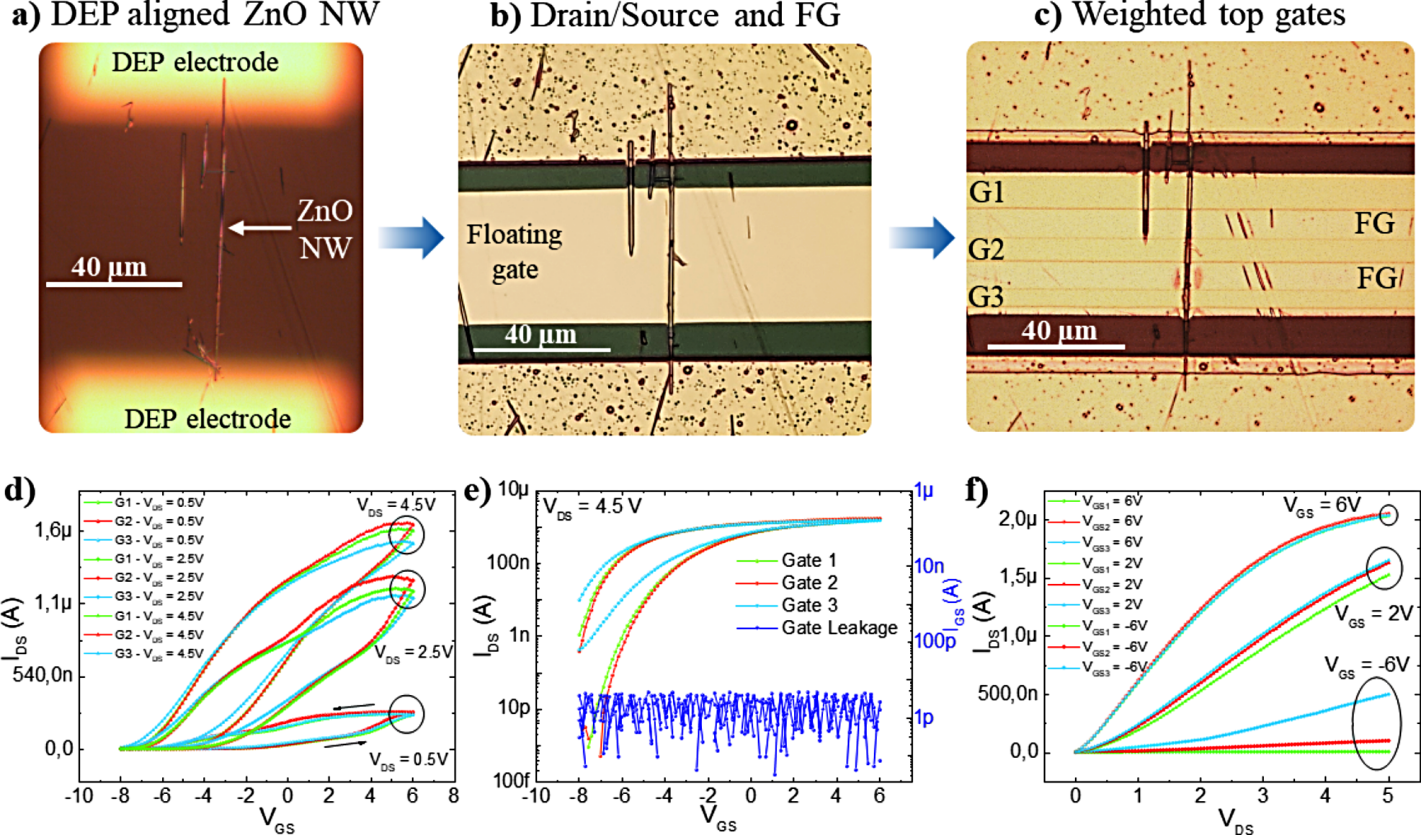
Fabrication and electrical characterisation of ZnO NW *ν*-FET: (**a**) Optical image of ZnO NW aligned using contactless dielectrophoretic assembly, (**b**) the intermediate stage of fabricated device showing deposited drain and source and floating gate electrode over the NW and 400 nm thick dielectric (SiN_x_), (**c**) fabricated device with 3 control gates, capacitively coupled to the floating gate with a thin 100 nm thick SiN_x_ in between. (**d**) Transfer characteristics with V_DS_ varying from 0.5 V to 4.5 V with a step of 2 V for each individual control gates (Gate 1 – Green, Gate 2 – Red, Gate 3 – Blue) with I_DS_ in linear scale and (**e**) transfer characteristics for each individual control gates and leakage current measured at V_DS_ 4.5 V with I_DS_ in log scale. (**f**) Output characteristics with V_DS_ swept from 0 to 5 V and V_GS_ varying from − 6 V, 2 V and 6 V for each individual control gate

Schematic of a typically fabricated single ZnO NW based *v*-FET device is shown in Fig. 1e, f. The optical micrographs displaying the images of a *v*-FET device at various fabrication stages are shown in Fig. 2a-c. The components of the device, from the bottom to the top, are: ZnO NW as active semiconducting channel on a polyimide (PI) substrate, the Ti/Au source and drain contacts, SiN_x_ floating gate dielectric layer (400 nm), the Ti/Au floating gate (10/100 nm), SiN_x_ top gate dielectric layer (100 nm), and the Ti/Au top control gates (10/100 nm). It is to note that the total dielectric thickness ranging from the NWs to the CGs is 500 nm. Single-crystalline, as-received commercially purchased ZnO NWs are dispersed in an iso-propanol (IPA) solution and deterministically integrated over a flexible PI substrate in an aligned manner using contactless DEP technique. DEP is a solution-based NW assembly approach offering attractive features for large area electronics such as precise positioning, high degree of orientation and cost effective [39]. In this work, we have modified the conventional DEP process for NW harvesting from an IPA solution. In conventional DEP process, using pre-defined microelectrode array (MEA) and applied voltage (AC or DC) signals, NWs are aligned over the metal electrodes resulting in a poor metal-semiconductor (MS) interface [40–42]. To improve the MS interface, another lithography step is needed each time the devices are fabricated. To avoid this, the modified DEP process is developed as follows: The DEP process was initiated by spin-coating a ∼ 15 μm thick PI substrate (PI2525 from HD microsystems™) over the carrier substrate (glass), where the DEP electrodes were pre-patterned by using photolithography process steps (see methods for details). The developed modified contactless DEP technique provides a carrier to process devices on flexible substrates (facilitates in aligning steps during lithography). It is to note that the spin-coated spin-on PI layer is thin enough to allow the electric field to propagate and attract NWs onto the substrate. Compared with the conventional DEP process, the MEA, used for the generation of electric field, does not come into a direct physical contact with the assembled nanomaterials and thus, could be re-utilised to assemble nanomaterials over different target device substrates. Consequently, the innovatively developed process reduces not only the number of manufacturing steps, but also the need for energy, time, consumables as well as the electronic wastage, a step towards circular electronics. Figure 2a shows an optical image of the aligned NWs between the buried DEP electrodes on PI. The DEP parameters are optimised using COMSOL simulation to achieve single ZnO NW alignment. The optimized DEP parameters are 50 V_PP_ and 500 kHz, at which sufficient forces (in the order of nN) are generated to attract, align, and place the NWs at the defined locations (see supporting information note 1 and Figure S1). The DEP parameters were optimised to capture single NW in the channel area, however, as can be seen from Fig. 2a, few short NWs were also attracted at the channel region. These short NWs were unable to make any contribution to the device conductance (not long enough to connect source-drain electrode). The aligned NW samples are further processed to construct *v*-FET devices. These include using conventional microfabrication steps for the deposition of gate dielectrics and source/drain/floating gate/control gates metal electrodes (Fig. 2b, c).

The fabricated top CGs cover the entire FG length (175 μm) with widths of ∼ 7 μm, ∼ 5.6 μm and ∼ 4.5 μm for CG1, CG2 and CG3, respectively. Considering a parallel plate capacitor relationship between the FG and the top CGs, the values for G1 (CG1), G2 (CG2) and G3 (CG3) are 9.7 × 10^− 13^ F, 7.7 × 10^− 13^ F and 6.2 × 10^− 13^ F, respectively. The electric potential at the floating gate (V_FG_) is expressed using the below Eq. 1 [23]:

1 $$\:{V}_{FG}=\:\frac{{\sum\:}_{i=0}^{\text{n}}{C}_{{w}_{i}\:}{V}_{{Gw}_{i}}}{{C}_{FG}+{\sum\:}_{i=0}^{\text{n}}{C}_{{w}_{i}\:}}+\:{V}_{Offset}$$

where C_wi_ is the capacitance between the individual gate and the floating gate and determines the weighing factor w_i_, V_Gwi_ is the voltage at each control gate, C_FG_ corresponds to the oxide capacitance between the FG and the NW and V_Offset_ is given by magnitude of the fixed oxide and interface trap charges. The synaptic weight (w_Gi_) of each control gate can be given by the coupling coefficients expressed by Eq. 2:

2 $$\:{w}_{Gi}=\frac{{C}_{i}}{{C}_{total}}$$

where,

3 $$\:{C}_{total}={C}_{FG}+\:\sum\:_{i=1}^{n}{C}_{i}$$

in which C_FG_ can be neglected due to its value being one order of magnitude lower (3 × 10^− 14^ F) when compared to the gate capacitances. The obtained synaptic weights for control gates G1 (w_G1_), G2 (w_G2_) and G3 (w_G3_) are 0.41, 0.33 and 0.26, respectively.

### Electrical characterisation of individual control gates

Before performing neural like functions, basic transistor tests such as transfer, and output curves were obtained for the fabricated *v*-FETs through biasing of the individual control gates. Figure 2d represents the transfer curves acquired from each control gate for different V_DS_ bias ranging from 0.5 V to 4.5 V with a 2 V step. In this test, only one of the multi control gates is biased while the others were kept floating (i.e., not probed). Irrespective of which gate is controlling the device, the transfer curves show a clear n-channel device behaviour where accumulation of charges with increasing positive voltages is shown. The device is normally-on (depletion type) giving output current at V_GS_ = 0 V because of unintentional n-type doping in ZnO NWs. Further, it is to note that a large hysteresis is observed in the transfer scans for all the gates. There could be many possible reasons for the observed hysteresis but most probably it is because of the charge trapping at the floating gate/dielectric interface and/or semiconductor/dielectric interface [43]. It is well-know that silicon nitride (SiN_x_) films deposited at low temperature using plasma enhanced chemical vapor deposition (PECVD) technique is a good charge storage material. More specifically, silicon-nitrogen dangling bonds (Si ≡ N), known as K centre defects, are the primary charge trapping defects present in the SiN_x_ films [44]. In the present work, we have deposited SiN_x_ films using PECVD at room temperature, which is expected to result in higher defect centres, and this could be the reason for observed hysteresis. We assume that the FG plays a dominant role in the charge trapping and thus, to the observed hysteresis. To validate the assumption, another device with no floating gate was fabricated and electrically characterised under similar conditions. The electrical data showed negligible hysteresis, further ratifying our assumption of the dominant role played by the floating gate in the observed large hysteresis (**supporting information Figure S2**). The curves shown in Fig. 2d exhibit hysteresis for all drain voltages, however, at low V_DS_ the observed hysteresis is larger than one observed at higher V_DS_ voltages. We have not probed the exact reason for this, but it is possible that at lower V_DS_ the lateral electric field strength is too weak compared with the vertical gate field strength which results in a higher charge trapping at the floating gate/dielectric interface and/or semiconductor/dielectric interface.

The spatiotemporal summation of synaptic inputs is essential for the transmission of information in biological systems, as neurons integrate inputs over time and ‘fire’ when a certain threshold is reached [37, 45]. This effect is emulated in our device through short-term plasticity, enabled by room temperature-deposited SiNx as the gate dielectric [36, 46]. The FG temporarily accumulates charge, mimicking the way a biological neuron’s membrane potential builds up until a firing threshold is surpassed. Additionally, such a spatiotemporal processing is highly effective in the somatosensory system for processing tactile information [47]. Through receptive fields, the spatial and temporal correlation of inputs enables perceptual object discrimination at the peripheral level. Implementing this capability in e-skins at the hardware level would be valuable for encoding tactile information and reducing the amount of data processed downstream [48].

Figure 2e shows the transfer curves at V_DS_ = 4.5 V in semi-logarithmic scale for better visualization of the effect of the gate width (synaptic weights) on modulation of the channel current. Key transistor performance metrics obtained from the transfer curves are on-state current (I_on_), off-state current (I_off_), current on/off ratio (I_on/off_), field effective mobility (µ_FE_), and subthreshold slope (s-s). The maximum I_on/off_ ratio can reach to 10^7^ (I_on_ = 1.7 uA) / I_off_ = 0.5 pA)) for both CG1 (G1) and CG2 (G2). Whereas the CG3 (G3) displayed poor I_on/off_ ratio (10^4^). The obtained s-s is approximately 833 mV/dec for G1 and G2, while G3 showed ∼ 1.7 V/dec. Comparing with the literature data on similar NW transistors, the obtained s-s values are higher, and this can be attributed to the presence of a comparatively thicker gate dielectric layer (500 nm) and to the fact that the control gates are not covering the NW channel entirety. Both the structural device parameters result in poor gate coupling with the semiconducting channel and thus, poor s-s for all control gates.

The field-effect mobility of the NW FETs is generally calculated using the Eq. 4 for a device operating in a linear regime, where g_m_ is the transconductance given by the Eq. 5, L_Ch_ is the channel length (∼ 50 μm) and C_FG_ is the floating gate capacitance. It is to note that the floating gate is not covering the channel entirely. Because of this the channel is divided in two regions: gated and ungated. In this scenario, the modified equation to calculate field effect mobility shown in the Eq. 6 (considering the dimensions of the ungated region), where L_C_ is the channel length, L_G_ the gated length, R_G_ the outer radius of the SiN_x_ coated NW and R_NW_ the radius of the NW. Equation 7 shows an expression to obtain C_FG_ for a typical omega-shaped NW top gated structure [49].

4 $$\:{\mu\:}_{FE}=\frac{{{L}_{Ch}}^{2}}{{C}_{FG}{V}_{DS}}{g}_{m}$$

5 $$\:{g}_{m}=\frac{\partial\:{I}_{DS}}{\partial\:{V}_{GS}}$$

6 $$\:{\mu\:}_{FE}=\frac{{L}_{C}{L}_{G}{R}_{G}}{{C}_{FG}{V}_{DS}{R}_{NW}}{g}_{m}$$

7 $$\:{C}_{FG}=\frac{2\pi\:{{\epsilon\:}_{0}\epsilon\:}_{r}{L}_{NW}^{2}}{\text{ln}\left(\frac{{R}_{g}}{{R}_{NW}}\right)}$$

Using the Eq. 7, the value of C_FG_ is approximately 3 × 10^− 14^ F with R_NW_ = 500 nm, R_G_ = 900 nm and ε_r_ = 9. Next, using the C_FG_ value and Eq. 6, the obtained peak linear mobilities (V_DS_ = 0.5 V) are 175, 176 and 142 cm^2^/Vs for control gates G1, G2 and G3, respectively. In the saturation regime, the mobilities decreased to 101, 126 and 94 cm^2^/Vs (V_DS_ = 2.5 V), and for V_DS_ = 4.5 V to 53, 52 and 48 cm^2^/Vs for G1, G2 and G3, respectively. The extracted peak mobility value (∼ 175 cm^2^/Vs for G1 and G2) is higher than most of the state-of-the-art ZnO NWs based FETs and this confirms the high quality of NWs and metal-semiconductor contacts formed. Further, it can be concluded that our contactless, DEP approach introduces very fewer surface impurities on the NWs. To further validate this, the output characteristics are performed and shown in Fig. 2f for all control gates. The data reveals ohmic contacts between the NW and Ti/Au metal contacts displaying near-linear increase in I_DS_ at low V_DS_ values, confirming clean interface between NW and the source/drain metal electrodes.

The effect of the synaptic weights (by the varying control gate widths) is evident from the electrical data shown in Fig. 2. When one of the control gates is swept from − 8 V to 6 V, while the other gates were held floating, the magnitude of current modulation is different. For instance, unlike the control gate G1 and G2 with higher widths, I_on_ is relatively high when G3 is at − 8 V. Meaning, the device cannot be turned OFF by G3 and can be considered as always ON (I_on/off_ ∼10^4^). However, when either of the gate G1 or G3 reaches − 8 V, the device reaches OFF state with I_on/off_ ratio of ∼ 10^7^. Similar effect of gate widths could be observed from the output scans as well. Such a device architecture can be considered as a two-input-one-output digital element enabling important logic gates such as AND (discussed in the later section). The effect of gate widths is further explored in the present work to mimic critical neural functions such as tuning the transistor threshold voltage, pre-synaptic input summation and short-term synaptic plasticity.

### Variable threshold control

**Fig. 3:**
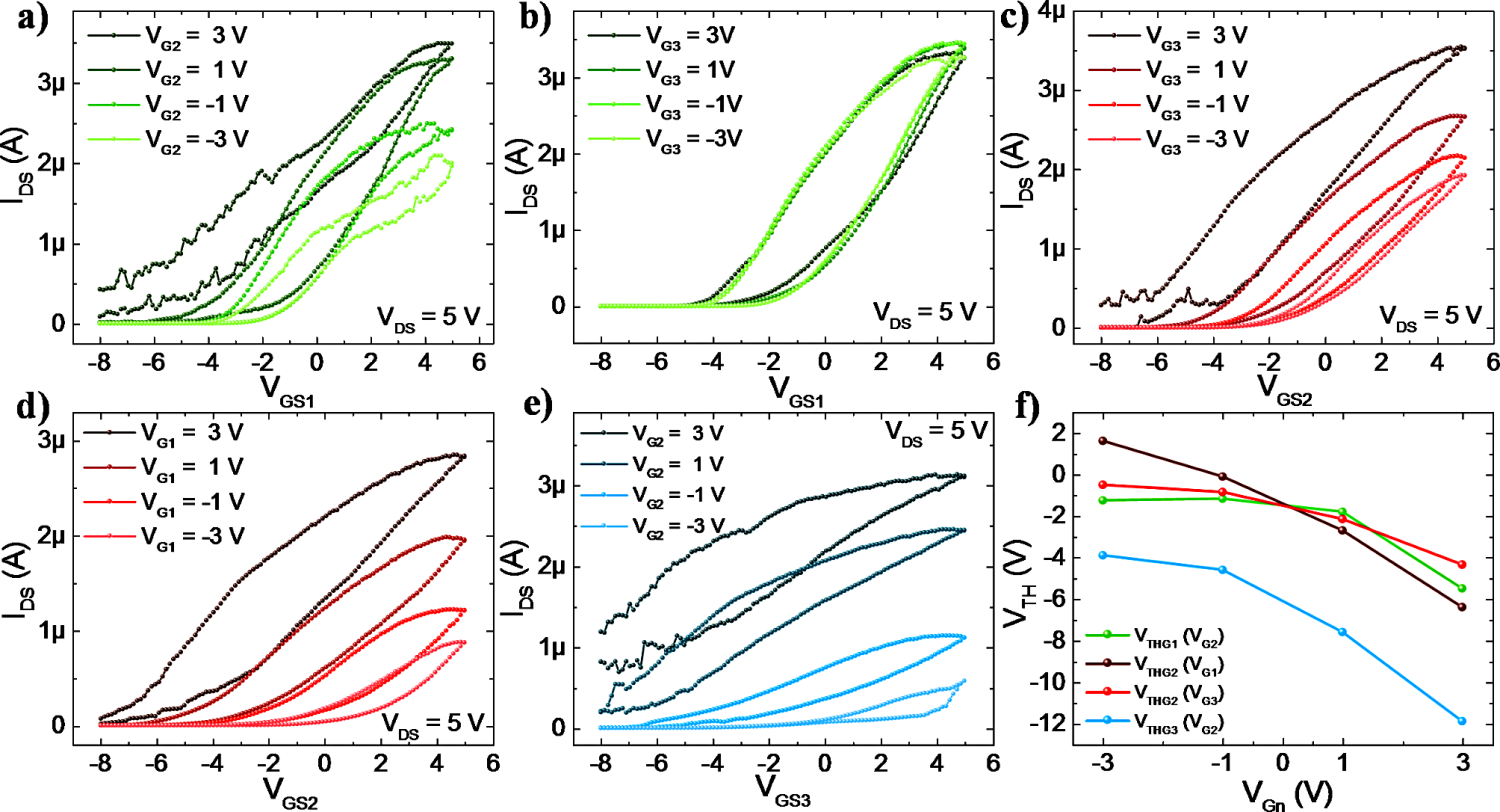
Transfer curve illustrating the threshold voltage shift seen by gate 1 (V_TH1_) when (**a**) gate 2 (G2) and (**b**) gate 3 (G3) is biased at fixed voltage, ranging from − 3 to 3 V with 2 V step, and with V_DS_ = 5 V at G1. Transfer curve illustrating the threshold voltage shift seen by gate 2 (V_TH2_) when (**c**) gate 3 (G3) is biased and (**d**) gate 1 (G1) is biased at fixed voltage, ranging from − 3 to 3 V with 2 V step, and with V_DS_ = 5 V at G2. (**e**) Transfer curve illustrating the threshold voltage shift seen by gate 3 (V_TH3_) when gate 2 (G2) is biased at fixed voltage, ranging from − 3 to 3 V with 2 V step with V_DS_ = 5 V at G3. (**f**) Threshold voltages seen by different gates while a second gate is under bias

One of the main capabilities of *v*-FETs is that they could modulate the transistor threshold voltage and on-current. The electrical data for these studies is presented in Fig. 3. The device threshold tuning is accomplished through the voltage-mode summation that defines the floating gate voltage (V_FB_) which spreads the electric field along the NW channel (see Eq. 1). A depletion-mode device is characterised by a negative threshold voltage, representing the necessary potential to deplete the channel, turning the device to an OFF state. The *v*-FET has a certain threshold voltage seen by the floating gate (V_TH*_). Where the device is turned off when the cumulative input voltages confer the floating gate enough negative potential to surpass the threshold of the device (V_FG_ < V_TH*_). Hence, each top gate will see a certain threshold voltage depending on the accumulated charges of the gate. Individually (only one probed gate), the threshold voltage is approximately V_TH_ = -3.4 V for both G1 and G2, and V_TH_ = -4.2 V for G3 at V_DS_ = 4.5 V. The lower weight of gate 3 is reflected on the higher negative voltage required to effectively deplete charges in the channel. It is to note that the threshold voltage is extracted using the linear extrapolation method, through the I_DS_–V_GS_ graph at the region where the linear extrapolation intercepts the I_DS_ = 0 at x-axis (V_GS_).

The V_TH_ expressed above is for individual gates, however, when more than one gate is probed the voltage summation effect at the floating gate due to the capacitive coupling will impact the threshold voltage of the device. A practical example of this can be described by selecting one gate as the input (voltage sweeping terminal) and the remaining as threshold control gates (with a fixed voltage). Considering G1 as the input gate, during a voltage sweep, a certain threshold voltage (V_TH1_) is observed, however, such V_TH1_ can be altered by applying a bias at G2 (V_G2_) which alters the V_FG_ and consequently, the V_TH1_. With this, the gate at fixed bias (G2) can be described as threshold control gate. To analyse the effectiveness of the voltage mode summation and how it affects the threshold voltage, series of tests are conducted. For these tests, the sweeping gate voltage (V_GSN_) defines the transistor operation wherein N denotes the gate number swept. The second gate with a fixed voltage performs the threshold voltage tuning function. Tests were performed by sweeping one of the control gates from − 8 to 5 V while the other is biased at a fixed voltage (-3, -1, 1 and 3 V) and the last remaining control gate floating (without any bias).

A clear shift in V_TH_ and I_on_ are observed while sweeping Gate 1 and Gate 2 is biased at a fixed voltage (Fig. 3a). Under negative voltages (V_G2_ = -1 and − 3), the threshold voltage would be expected to shift to the right as less negative voltage is required to deplete the channel. However, the effect is negligible as the threshold voltage remains similar but a significant large change on the I_ON_ is observed. One possible explanation is the fact that the weight of G1 (*w* = 0.44) is higher than G2 (*w* = 0.33), showing its dominating effect over the channel control. Nevertheless, when V_G2_ was biased at a positive 3 V, a strong accumulation of charges in the semiconductor channel occurred, shifting the V_TH1_ to the more negative voltages. Similar test was performed while sweeping Gate 1 and Gate 3 is biased at a fixed voltage (Fig. 3b). The obtained results show negligible changes in threshold voltage and on-current. This is expected as G3 has the lowest synaptic weight (0.26).

The effect of higher synaptic weight presented by G1 in threshold and on-current tuning of G2 is elucidated from the data shown in Fig. 3c and d. The threshold varied almost linearly (R^2^ = 0.95) with the varying V_G1_ proving the dominating effect and voltage summation at the FG (dark red curve in Fig. 3f). The V_THG2_ increased from − 6.4 to 1.6 when V_G1_ is 3 V and − 3 V, respectively. A similar effect was observed when we biased G3. The V_THG2_ shifts to the left (negative) when we applied positive gate voltages at the G3 - the total shift ranges from − 0.5 to -4.3 with V_G3_ at -3 V and 3 V, respectively. The total shift of G2 threshold (ΔV_THG2_) when we biased G1 and G3 is approximately 8 V and 4.8 V, respectively. This follows the weight relation as w_1_ (0.41) > w_3_ (0.26). The weight effect can be seen in Fig. 3f, where the V_THG2_ is plotted against the applied bias at a secondary gate. Due to the higher weight of G1, the slope (V_TH_ vs. V_GN_) is higher which demonstrates a larger threshold shift and voltage mode summation effect at the FG. Finally, the threshold voltage shift seen by G3 is analysed by biasing G2 (Fig. 3e). As it is expected, the higher synaptic weight of G2 demonstrates a strong dominance over G3. For instance, when the G2 is positively biased with 3 V, it is not possible to deplete the channel. The changes in V_TH_ (Fig. 3f) are summarised in Table 1. From this data, higher synaptic weights dominate over lower weights to modulating the source/drain current. Further, the threshold shifts confirm the voltage mode summation, which is the foundation of potential spatial summation of synaptic inputs.

**Table 1:** Threshold voltage shift relationship between 2 gates. *Signifies extrapolated data

V_BIAS_		V_TH_ (Gate 1)	V_TH_ (Gate 2)	V_TH_ (Gate 3)
Gate 1	-3	-	1.6	-
	-1	-	-0.1	-
	1	-	-2.7	-
	3	-	-6.4	-
Gate 2	-3	-1.4	-	-3.9
	-1	-1.4	-	-4.6
	1	-4.4	-	-7.6*
	3	-8.3	-	-11.9*
Gate 3	-3	-	-0.5	-
	-1	-	-0.8	-
	1	-	-2.1	-
	3	-	-4.3	-

### Synaptic characteristics of *v*-FET

**Fig. 4:**
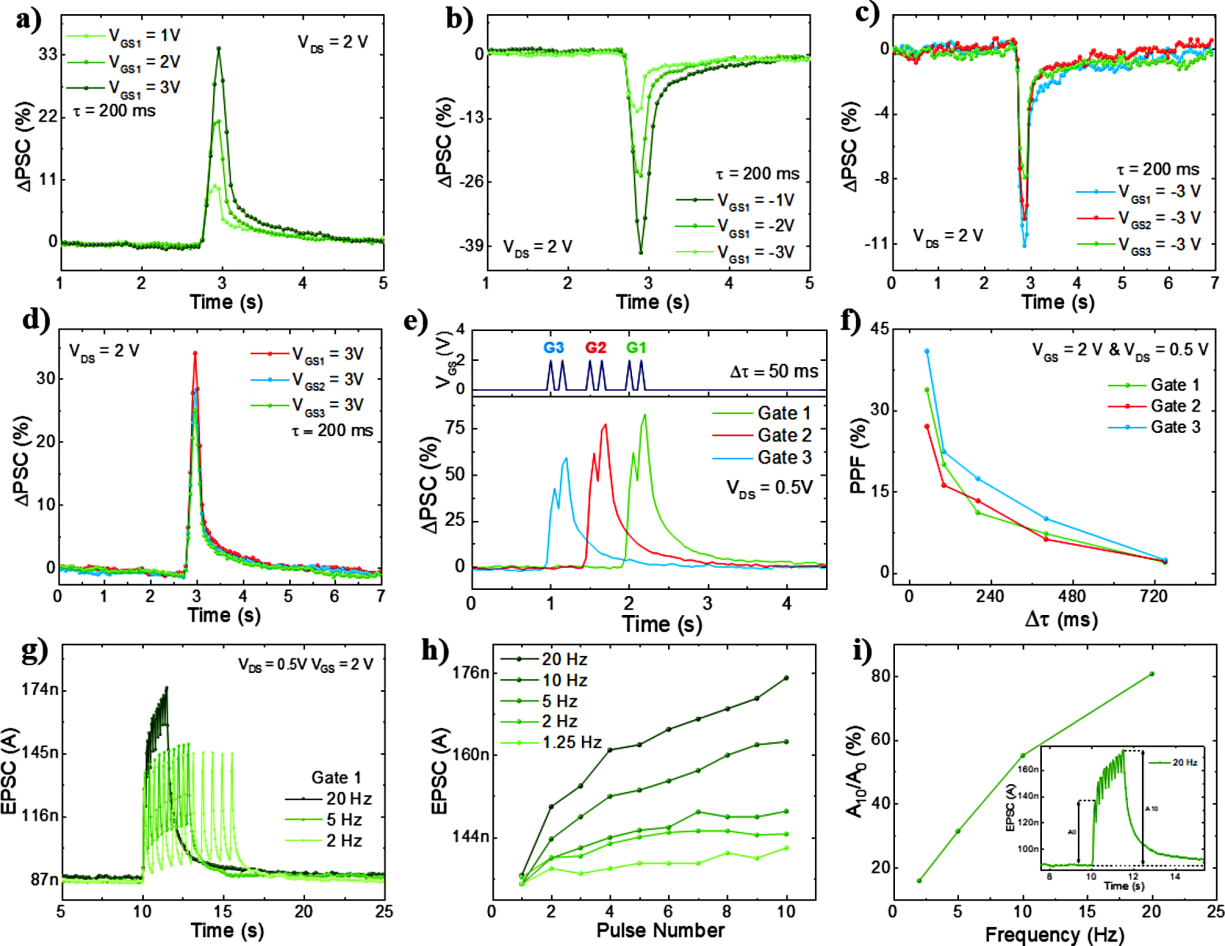
(**a**) post-synaptic current modulation with 200 ms pulse with amplitudes ranging from 1 V to 3 V on control gate 1. (**b**) Post-synaptic current modulation with 200 ms pulse with amplitudes ranging from − 1 V to -3 V on control gate 1. (**c**) Comparison between the post-synaptic current with 200 ms pulse at -3 V applied on all three control gates. (**d**) Comparison between the post-synaptic current with 200 ms pulse at 3 V applied on all three control gates. (**e**) ΔPSC triggered by an input pair with 50 ms delay for the 3 gates interdependently at V_DS_ = 0.5 V. (**f**) PPF index (%) for each gate as a function of the time interval (Δt). (**g**) EPSC curve showing high-pass frequency filtering characteristics for gate 1. (**h**) EPSC modulation of the device to the increasingly pulse numbers with varying frequency. (**i**) Current gain (A_10_/A_0_) in percentage provided by spike train of 10 pulses (inset) at various frequencies

Owing to the voltage summation at FG and hysteresis effect exhibited in the electrical characterisation, spatial and temporal dynamics are emulated in the fabricated device. The performance is analysed by applying spike-based input voltages on the various gates, to understand how the temporal and spatial corelation of inputs modulate the output current. The spike-based input measurement is crucial when considering integration of the device in spiking neural networks, as the device could interface slow-adapting and fast-adapting spike-based sensors (spike-based output) or the outputs of digitalized pulsed stimulus [50, 51]. The voltage applied at the multiple control gates of the *v*-FET are defined as pre-synaptic input (V_pre_), where the measuring I_DS_ is described as post-synaptic current (PSC). In an analogy with the biological neuron, the top gates can be seen as pre-synaptic membranes and drain/source as post-synaptic membranes. The resulting variation in current after the applied spike (ΔPSC) is expressed by using Eq. 8:

8 $$\:{\Delta\:}\text{P}\text{S}\text{C}\:\left(\text{\%}\right)=\frac{{\text{I}}_{\text{P}\text{e}\text{a}\text{k}}-{I}_{0}}{{I}_{0}}\times\:100$$

where, I_Peak_ is the peak current of the stimulus and I_0_ the base current before the stimulus. The spike tests are started by applying positive (excitatory) and negative (inhibitory) inputs on the control gates. The applied inputs are electrical pulses with 200 ms duration at gate voltages ranging from 1 V to 3 V, with V_DS_ = 2 V. As observed in Fig. 4a, an excitatory pulse (positive) is applied to G1 resulting in the rise of the PSC (for n-channel FETs) and gradually decay of current to the initial values. Such effect is analogous to the excitatory modulation effect in the biological synapse and is named as ‘excitatory post-synaptic current (EPSC)’. As depicted in the electrical curve, higher amplitudes voltage leads to a larger peak current giving the ΔPSC of 10%, 21% and 34% with 1 V, 2 V and 3 V inputs, respectively. On the contrary, the PSC abruptly decreases and gradually recovers after applying a negative pulse, mimicking IPSC (inhibitory post synaptic current). The inhibition is emulated by applying similar polarised voltages (-1, -2 and − 3 V), leading to a ΔPSC of -11%, -24% and − 40% (Fig. 4b). Additionally, higher amplitudes lead to higher decays and charge trapping effects, which is inherent to all the CGs (**supporting information Figure S3**). The synaptic weight effect is elucidated by comparing the same input at the independent CGs. When a V_pre_ of + 3 V is applied to the CGs (Fig. 4d), the ΔPSC is 34% (G1), 28% (G2) and 24% (G3). Interestingly, the lower ΔPSC of G2 and G3 when compared with G1 is coherent with the defined weight relation, resulting from the fabricated gate widths and consequently different capacitive coupling coefficients (w_G1_ > w_G2_ > w_G3_).

Exploiting the floating gate charge-trapping effect, paired pulse facilitation (PPF), an essential short-term plasticity effect inherent of synapses, is demonstrated. Such effect plays an important role in temporal information processing, described as the enhancement of ΔPSC when a second spike closely follows the previous spike in the millisecond (ms) range and is given by Eq. 9:

9 $$\:\text{P}\text{P}\text{F}\:\left(\text{\%}\right)={\text{A}}_{2}/{\text{A}}_{1}\times\:100$$

with A_1_ and A_2_ defined as the peak current at the first and second stimulation. The PPF is demonstrated by applying two voltage pulses (2 V) at the same gate with Δτ = 50 ms and V_DS_ fixed at 0.5 V. The tests are conducted at low drain voltage given that higher hysteresis value was obtained when biased at lower drain voltage, resulting in enhanced memory effects. The obtained responses (Fig. 4e) evidently show short-term plasticity for all the gates, where G1, G2 and G3 demonstrate a ΔPSC with the second spike of 83%, 78% and 60%, respectively.

Neural systems are highly adaptable due to the training of synaptic weights, however, when these synaptic weights are hardwired, adaptability is hindered, as in the present case. Future designs could integrate memristive elements with the *v*-FETs (1 M-1T configuration) which would offer non-volatile long-term plasticity enabling learning and memory functions while performing spatiotemporal integration of inputs. These building blocks are promising for advanced neuromorphic systems, as they establish the initial levels of the neural network directly at the hardware level, enabling systems such as e-skins to greatly benefit from these encoding techniques [23].

The PPF is extracted and plotted for all the CGs (Fig. 4f), where the index quickly decreases with the increase of time interval between pulses. This is because the charge accumulation effect fades away due to the release of the trapped charges with time. Further experiments were conducted by applying spike trains with 10 pulses. G1 was selected for such tests due to the higher modulation of ΔPSC (given by the higher weight). Figure 4g shows the device response for positive spike trains (10 pulses), applied at different frequencies (20, 5, and 2 Hz). It is evident that high frequency signals tend to accumulate large number of charges and grant higher peak currents, when compared to low frequency signals. Similar effect is observed for inhibiting inputs at various frequencies as shown in **supporting information Figure S4**.

The observed short-term potentiation and inhibition can be represented as a high and low pass filter, respectively. This behaviour is characterised by the facilitation and blocking of information transmission, inherent to biological synapses, where high frequency signals are transmitted and low-frequency signals are blocked [52]. In Fig. 4h, the peak current is shown corresponds to each pulse number for various frequencies. The peak output current notably increasing with the number of pulses for higher frequencies (> 10 Hz). The extracted frequency gain, defined as the ratio between the tenth peak (A_10_) and the first peak (A_1_), is 15% for pulses applied at 1.25 Hz which then increases to 80% for pulses at 20 Hz. Indicating that the *v*-FET can function as a dynamic high-pass filter (Fig. 4i).

**Fig. 5:**
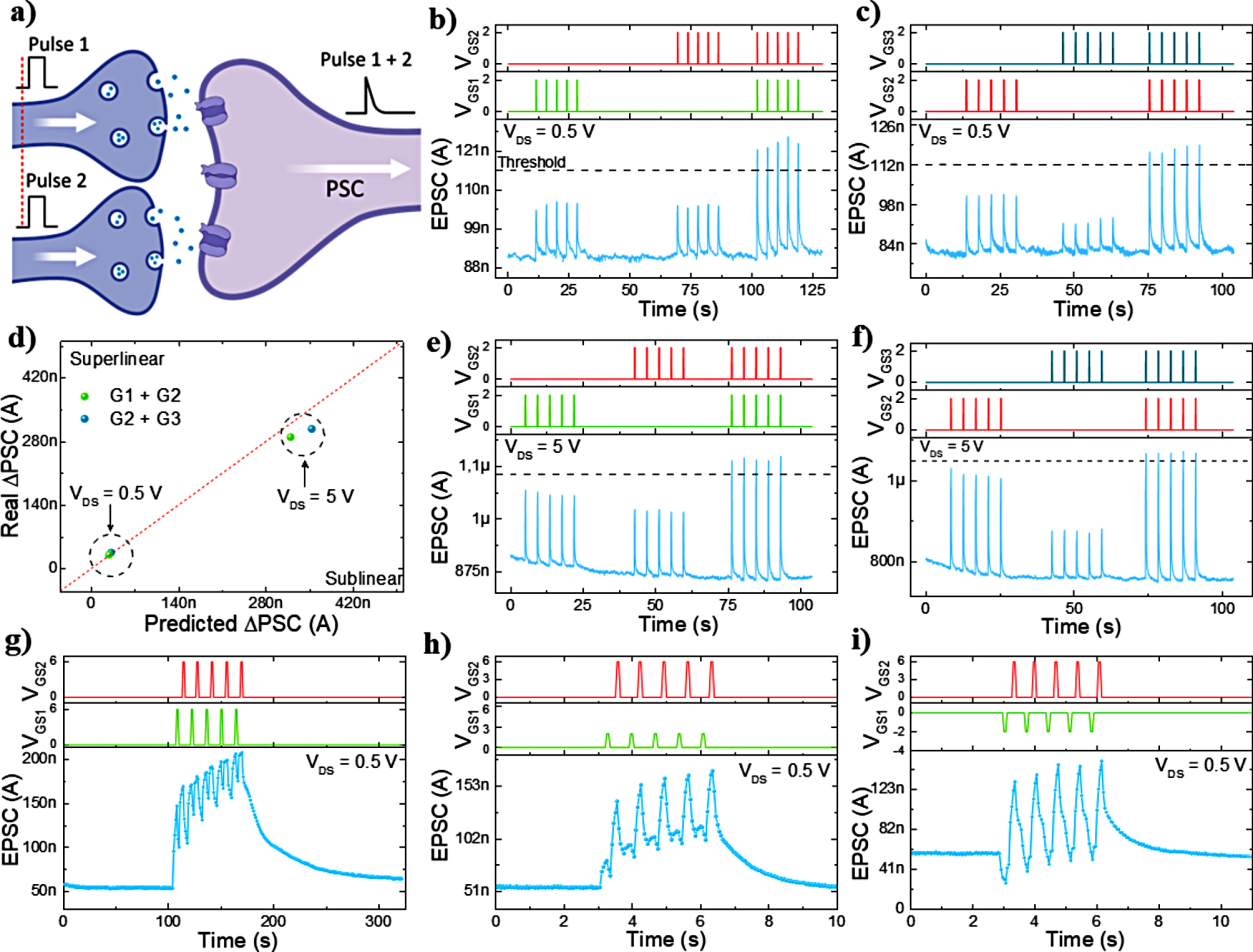
**(a)** Schematic of biological dendritic integration of two presynaptic inputs on the post synaptic neuron. **(b)** EPSC modulation of G1 and G2 dual-terminal gate input with 200ms spikes at 2 V and V_DS_ = 0.5 V. **(c)** EPSC modulation of G2 and G3 dual-terminal gate input with 200ms spikes at 2 V and V_DS_ = 0.5 V. **(d)** The measured sum current plotted as a function of the arithmetic sum current for the gate combinations at V_DS_ = 0.5 V and 5 V. **(e)** EPSC modulation of G1 and G2 dual-terminal gate input with 200ms spikes at 2 V and V_DS_ = 5 V. **(f)** EPSC modulation of G2 and G3 dual-terminal gate input with 200ms spikes at 2 V and V_DS_ = 5 V. **(g)** EPSC modulation by applying an asynchronous spike train with G1 and G2 biased at equal amplitude (6 V) at V_DS_ = 0.5 V. **(h)** EPSC modulation by applying an asynchronous spike train with different amplitude, G1 (2 V) and G2 (6 V) at V_DS_ = 0.5 V. **(i)** EPSC modulation by applying an asynchronous spike train with inhibitory (G1 = -2 V) and excitatory (G2 = 6 V) inputs at V_DS_ = 0.5 V

Given the results of spike tests at the individual gates, it is evident that the device possesses an intrinsic short-term memory when stimulated by pre-synaptic pulses. Benefiting from the floating-gate, the *v*-NWFET can emulate spatial summation functions (dendritic integration of a neuron), by summing input pulses simultaneously applied to the different control gates, analogous to the synaptic integration of a neuron as shown in Fig. 5a. The linear summation is evaluated by applying a set of pulses at a fixed amplitude (2 V) and drain voltage of 0.5 V and 5 V. It is to note that experimental results for other biasing combinations are shown in **supporting information Figure S5 and S6**. The experimental details for the results shown in Fig. 5 consists of applying 5 voltage pulses (200 ms) at 0.25 Hz to each gate separately, followed by triggering of both gates simultaneously. With this it is possible to analyse the ΔPSC of each gate individually and when the signal is combined. The EPSC responses are obtained when V_pre_ pulses (2V_GS_ and 0.5V_DS_) are applied to G1 and G2 (Fig. 5b), and G2 and G3 (Fig. 5c). Similar levels of ΔPSC are monitored for all the spikes inside each pulse set when applied to individual control gates, confirming a robust and repeatable response of the device. Precisely, a ΔPSC of 0.9 µA to 1.04 µA for G1 is obtained while G2 modulates the current from 0.91 µA to 1.04 µA. This provided a ΔPSC of ≈ 15.7% for G1 and ≈ 15.5% for G2. The change is equal to both gates which is not consistent with the weight variation of the individual spike tests conducted above, as G1 is expected to give higher PSC change than G2. Nevertheless, the combined gate input (G1 + G2) exhibited a ΔPSC from 0.9 µA to 1.21 µA which represents a ΔEPSC of ≈ 34.5%. Similarly, testing of CG 2 and 3 yields a ΔPSC of ∼ 26% (G2) while G3 exhibits a ΔPSC of 13% (Fig. 5c). The combined input showed a total change of 44.9%. Similar tests were performed when different V_pre_ pulses (2V_GS_ and 5V_DS_) are applied to the same gate combinations - obtained results are shown in Fig. 5e and f. The results for different V_pre_ applied i.e., 2V_GS_ and 0.5V_DS_ and 2V_GS_ and 5V_DS_ are summarised in Fig. 5d. The data for V_DS_ = 0.5 V shows a near-linear summation of EPSC. Precisely, a variation of ∼ 3.3% from the expected output is observed when pulses are applied to G1 and G2. Whereas a ∼ 5.5% variation is monitored when pulses are applied to G2 and G3. Similarly, near-linear summations were observed for the gate combinations at V_DS_ = 5 V, displaying a ∼-2.4% and ∼ 5.7% variation for G1,2 and G2,3. The small deviation from an ideal linear behaviour could be because of the high dielectric thickness (∼ 500 nm) and to the fact that the CGs are not covering the channel entirely. Both reasons could lead to poor gate coupling with the semiconducting NW channel. Furthermore, at the nanoscale, it is challenging to realise ideal metal-semiconductor (MS) and/or semiconductor/dielectric interfaces, especially with non-conformal dielectrics, as deposited in the present study. It is to note that using a *v*-FET device, logics can be simulated when both gates are simultaneously triggered. The binary input voltage applied at G1 and G2, for instance, allows the output current to reach a pre-defined threshold (see dotted line in Fig. 5b-c and e-f), showing that the *v*-FET device exhibits a typical AND logic gate behaviour.

Lastly, another set of experiment are conducted by applying asynchronized spike pulses, contrary to tests performed earlier, where the spikes were applied at the same time (synchronized). However, in biological systems asynchronized spikes are more likely to occur, making more realistic to analyse the device performance with asynchronous spike trains [53] Fig. 5g-i show the monitored response from the *v*-FET when both control gates are stimulated with similar spike trains of 200ms width and 700ms delay between spikes (the spike trains are separated by 300ms between both gates). The data is obtained for a fixed V_DS_ = 0.5 V. The data for V_DS_ = 5 V is shown in **supporting information Figure S7**. Various voltage combinations were performed to simulate excitatory and inhibitory behaviours. Excitatory synaptic integration is observed when both gates are triggered with positive voltage. Through accumulation of inputs, the device output current increases from 53nA to 208nA after applying the spike train to G1,2 with a magnitude of 6 V, representing a change of 392% (Fig. 5g). However, when one of the inputs is reduced to 2 V (G1 = 2 V, G2 = 6 V), the device exhibits a change of 300% given the smaller gate bias voltage (Fig. 5h). Moreover, when G1 receives an inhibitory stimulation (-2 V) and G2 an excitatory stimulation (6 V) (Fig. 5i), the device has a total change of 260% proving the inhibition of signals at the floating gate level through subtraction of inputs.

## Conclusions

In conclusion, we have designed and fabricated ZnO NW-based *v*-FET devices on flexible substrate and demonstrated their capability for biomimicking spatial and temporal summation function of a biological neuron. The nanoscale functional electronic layers (ZnO NWs) were assembled at selected locations using contactless DEP. This modified DEP approach for NW assembly is advantageous for low-cost, large-scale and large area fabrication of *v*-FET devices with a flexible form factor. The fabricated *v*-FETs are designed to have multiple control gates (CG) with varying widths which are capacitively coupled with the floating gate (FG). The capacitance of the overlapping areas between individual CGs and the FG determines the synaptic weights. The electrical characterisation performed after biasing (at low 0.5 V drain bias) each CG of *v*-FETs showed high effective peak mobilities in linear regime of 175 (control Gate 1), 176 (control Gate 2) and 142 cm^2^/Vs (control Gate 3) confirming high electronic grade quality of the assembled nanoscale electronic layers. The smallest gate width (4.5 μm) yielded lowest device mobility (142 cm^2^/Vs) due to the lowest synaptic weight. By simultaneously biasing two CGs, the fabricated *v*-FET devices were shown to mimic neuronal behaviour including threshold modulation, dendritic summation, and stable short-term memory. As an example, the total shift of G2 threshold voltage when we biased G1 and G3 separately is approximately 8 V and 4.8 V, respectively. This follows the weight relation as G1 weight w_1_ (0.41) > G3 weight w_3_ (0.26). This means, with a carefully selected operational point, the information stored in the FG of the *v*-FETs could be used to perform the computational logic. The synaptic weight effect is further elucidated by comparing the calculated ΔPSC for the same input voltage (V_pre_) applied at the independent CGs. When a V_pre_ of + 3 V is applied to the CGs, the ΔPSC is 34% (G1), 28% (G2) and 24% (G3). Interestingly, the lower ΔPSC of G2 and G3 when compared with G1 is coherent with the defined weight relation, resulting from the fabricated gate widths and consequently different capacitive coupling coefficients (w_G1_ > w_G2_ > w_G3_). The synaptic weight relation of CGs was further confirmed by the short-term plasticity for all the CGs, where G1, G2 and G3 demonstrate a ΔPSC of 83%, 78% and 60%, respectively. Finally, analogous to the synaptic integration of a neuron, spatial summation function was mimicked for both synchronous and asynchronous input pulses applied to the different CG combinations. The developed low-temperature fabrication process for *v*-FETs makes the presented approach promising for large area *v*-skin. ZnO NWs were chosen as active channel material due to their attractive electronic properties, solution processability and commercial availability. In future, other material candidates such as NWs of In_2_O_3_, SnO_2_, Si etc. as well as other device designs such as having multi-material dielectric stack for more controlled charge trapping, will be investigated. The large-scale fabrication of the demonstrated *v*-FET device could lead to a new brain intelligence computational architecture that can be adapted to develop next generation solutions such as neuromorphic skin (*v*-skin), which find use in a range of emerging applications such as prosthetics, robotics, human-machine interactions, smart packaging, wearables, and healthcare patches etc.

## Methods

### Dielectrophoretic substrate stack preparation

The DEP NW alignment method is based on 3 steps, first by the fabrication of microelectrode arrays (MEA) using conventional photolithography, then a flexible layer of polyimide is spun on the top of MEA. The polyimide layer acts as the substrate on which nanowires are aligned. The process is finished by preparing the nanowire dispersion and consequent electric-field induced alignment. First, a glass slide was cleaned in ultrasonication bath and subsequentially in acetone, iso-propanol, and DI water. The photoresist was exposed to UV, developed, and then treated with oxygen plasma (150 W for 30 s). The process was completed by depositing 10 nm Ti and 90 nm of Au films through e-beam deposition followed by lift-off. The DEP MEA has a 75 μm gap and 75 μm width. Secondly, PI2525 from HD microsystems^™^ was spun over the MEA. The spinning conditions were 30s at 500 rpm followed by 2000 rpm for 1 min. A second layer was spun after semi-curing the first layer at 120ºC for 2 min. Then, a hard baking step was carried out at 300ºC for 2 h.

### NW dispersion and dielectrophoretic alignment

The as-purchased ZnO NWs from Novarials are used to prepare a dispersion. Briefly, 0.001 g of cluster powder was added in a 10 mL of IPA followed by 3 min sonication to disperse the NW cluster and obtain a uniform dispersion. The NW assembly process include a dipping of the prepared substrate stack at 1 mm/s on a container having NW dispersion. An AC signal of 50 V_PP_ and 500 kHz was applied during the submersion of the substrate stack using a signal generator WaveStation 3082 from Teledyne LeCroy and an amplifier A400DI from FLC electronics.

### Device fabrication

The device was fabricated through following conventional photolithography steps. A thermal treatment of 150ºC for 30 min was performed after NW alignment to remove organic solvents from the surface of the NWs prior to fabrication steps. Then, LOR (Lift-off resist) was spun for 30s at 3000 rpm followed by soft baking at 150ºC for 2 min. Secondly, S1818 photoresist was spun for 30s at 3000 rpm finished with soft baking at 120ºC. The bi-stack resist layer was UV exposed for 6s and developed with MF319 for 2 min. Before metallization, an Oxygen plasma was performed at 100 W for 1 min. The drain and source metal contacts were deposited by e-beam evaporation consisting of 10 nm Ti and 90 nm of Au, finished by lift-off within acetone. The first dielectric layer consists of 400 nm CVD deposited SiN_X_ at room temperature. The floating gate is patterned and deposited following the previous procedure with the metal stack of Ti/Au with 10/40nm thickness. The top gate dielectric layer is 100 nm thick SiN_X_ layer, and the multiple gates have thickness of 10/90 nm Ti/Au. The device was finished by etching the drain and source region through reactive ion etching.

### Electrical characterization

Electrical characterizations were performed in the ambient environment using Cascade Micro-tech Auto-guard probe station interfaced to a semiconductor parameter analyzer (B1500A, Agilent).

## Electronic supplementary material

Below is the link to the electronic supplementary material.

Supplementary Material 1

## Data Availability

The data that support the findings of this study are available in the supplementary material of this article.
