# Supplementary material for: Multi-gate neuron-like transistors based on ensembles of aligned nanowires on flexible substrates
Source: Nano Converg. 2025 Jan 18;12:2. doi: 10.1186/s40580-024-00472-z (PMC11741959; doi:10.1186/s40580-024-00472-z)
Supplement: Supplementary file 1 — Supplementary Material 1 [file 40580_2024_472_MOESM1_ESM.docx]

**Multi-Gate Neuron-like Transistors based on Ensembles of Aligned Nanowires on Flexible Substrates**

João Neto^1^, Abhishek Singh Dahiya^2^ and Ravinder Dahiya^2^*

^1^James Watt School of Engineering, University of Glasgow, Glasgow, G12 8QQ, UK

^2^Bendable Electronics and Sustainable Technologies (BEST) Group, Electrical and Computer Engineering Department, Northeastern University, Boston, MA 02115, USA

*Correspondence to: [r.dahiya@northeastern.edu](mailto:Ravinder.Dahiya@glasgow.ac.uk)

**Supporting Note 1**

**DEP simulations**

The DEP force (F_DEP_) experienced by a high aspect-ratio oblate spheroid (one-dimensional nanomaterials) particle can be estimated by the following expression^[1]^ (eq.1):

$F_{\mathrm{DEP}}=\frac{2\pi\left( ab^{2} \right)}{3}\varepsilon_{m}\mathrm{Re} \left[ K\left( \omega\right) \right]\nabla E^{2}$ (1)

where ∇E is the gradient of the electric field, ε_m_ is permittivity of the medium, a is the long and b is the short radius of the ellipsoidal particle, respectively and Re [K (ω)] is given by the real part of the complex Clausius-Mossotti factor K(ω). The Clausius-Mossotti factor is divided into 2 components defined by the short and long axis, where the short axis corresponds to the radius which is responsible by the translation of the NW and the long axis by the length of the NW, which is related to the alignment of the structure with the electric field lines.^[2]^ Both components are approximated through the following expressions^[2]^ (eq. 2-4) through MATLAB. The zinc oxide NW dielectric properties were defined as ε_r_ = 6.5 ^[3]^ and σ = 480 S/m ^[4]^, with length of 100 µm and 500 nm radius, the medium solvent is isopropyl alcohol where the dielectric properties are defined as ε_r_ = 18 and σ = 6x10^-6^ S/m.

$$F_{CM-Long}= \frac{\varepsilon_{P}^{*}-\varepsilon_{m}^{*}}{\varepsilon_{m}^{*}} \left( 2 \right) \wedge F_{CM-Short}=2 \frac{\varepsilon_{P}^{*}-\varepsilon_{m}^{*}}{\varepsilon_{P}^{*}+2\varepsilon_{m}^{*}} (3)$$

$\varepsilon^{*}=\varepsilon_{p}-j\frac{\sigma}{\omega}$ (4)

The DEP force is estimated by integrating the DEP equation (eq.1) into COMSOL, the model is built with a glass carrier where the metal electrodes are defined on top (75 µm gap), the top layer is polyimide 10 µm thick which interface the liquid medium and the metal electrodes. The DEP force is plotted for the x and y axis in the liquid medium (IPA) giving values ≈ 30 nN in the region 100 µm above the superstrate (y = 110 µm of fig. S1b and c) with an AC signal of 50 V_PP_ at 500 kHz.


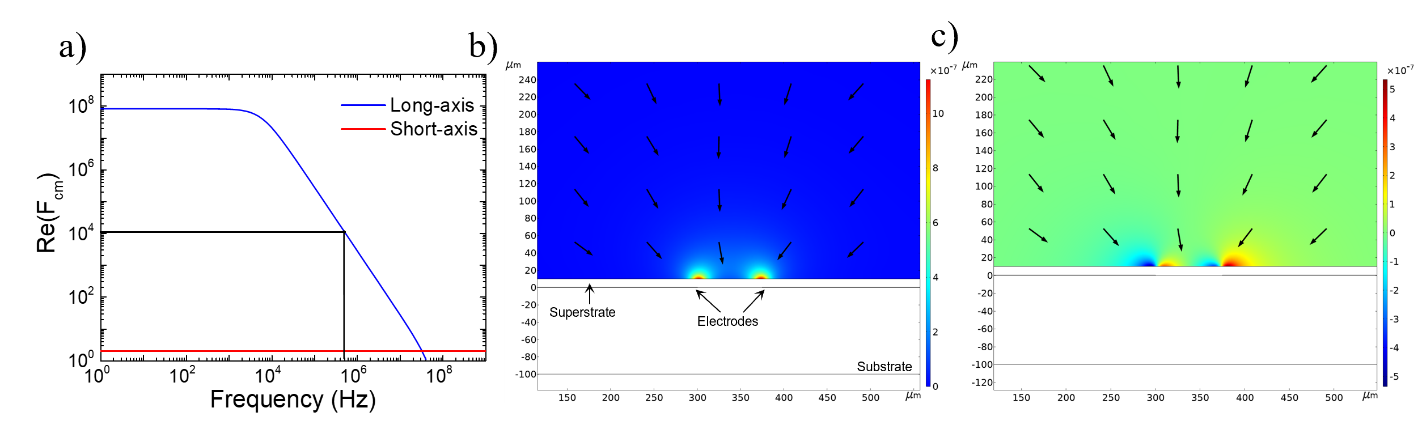


**Figure S1**. (a) Clausius-Mossotti factor estimation. b) DEP force in the y axis. c) DEP force in the x axis.


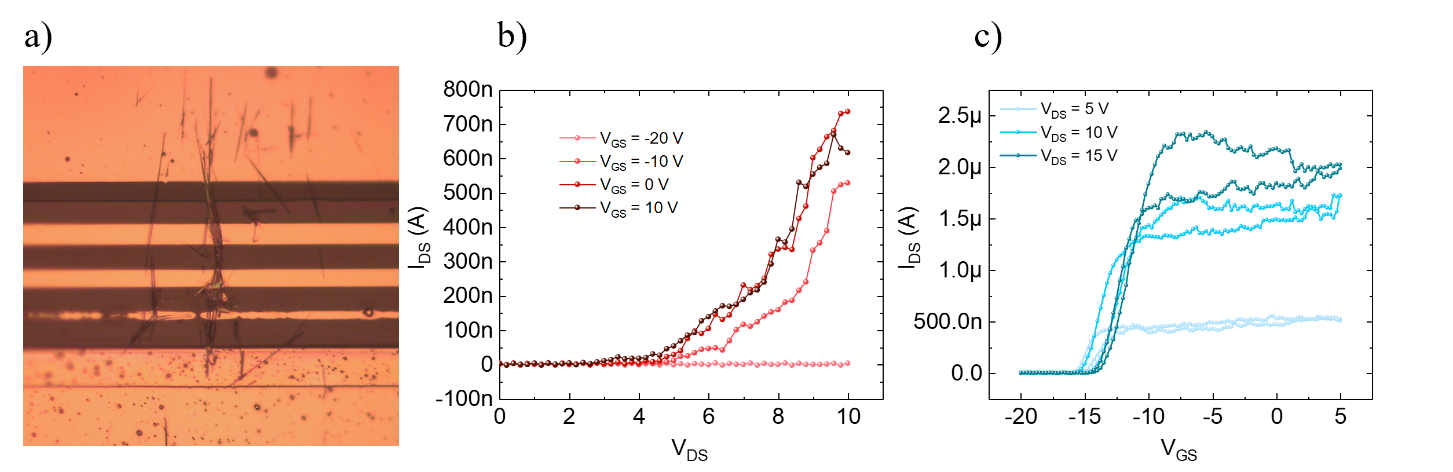


**Figure S2** – a) Optical image of the fabricated NW *v*-FET without the floating gate, b) Gate 1 output curve, and c) Gate 1 Transfer curve.


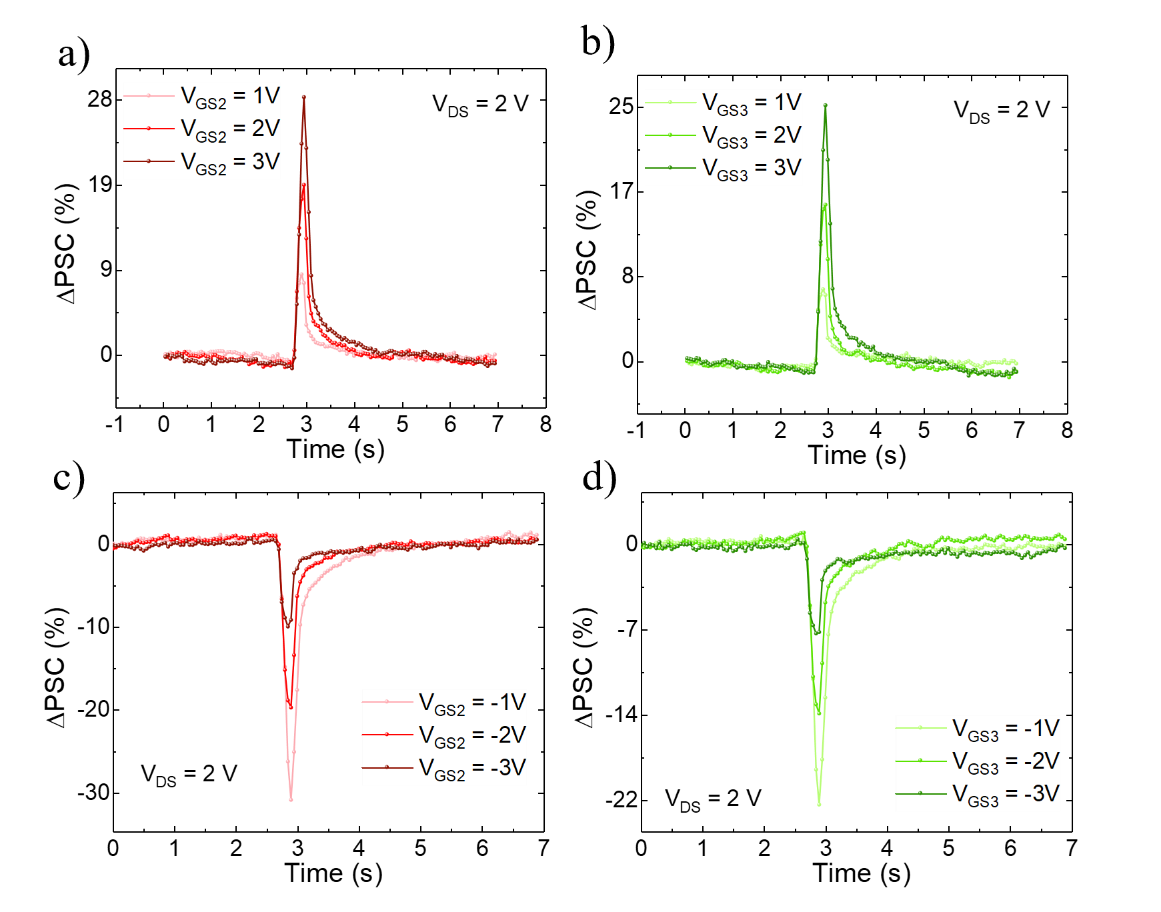


**Figure S3** – Positive and negative amplitude tests performed on gate 2 and gate 3.


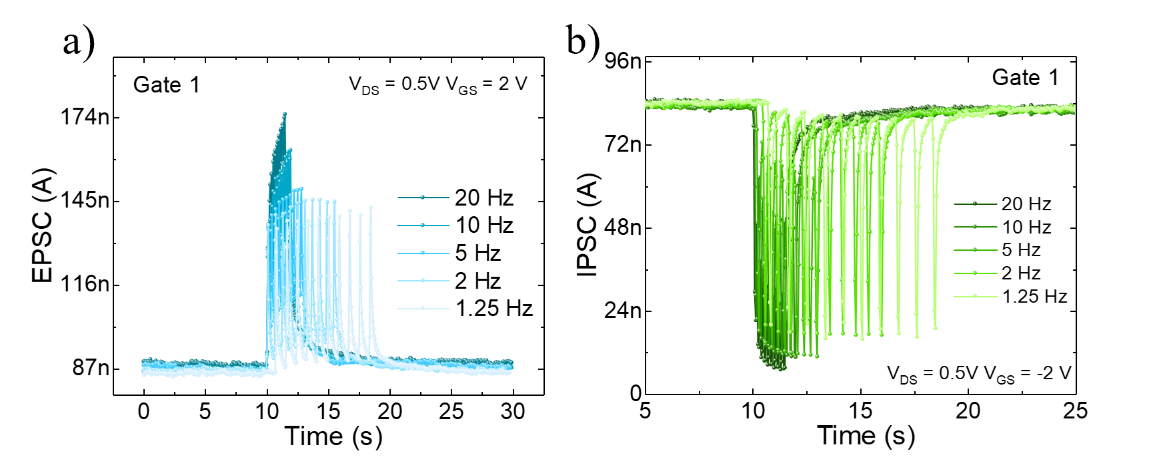


**Figure S4**. Spike train pulses applied at G1 with a) positive polarity, and b) negative polarity.


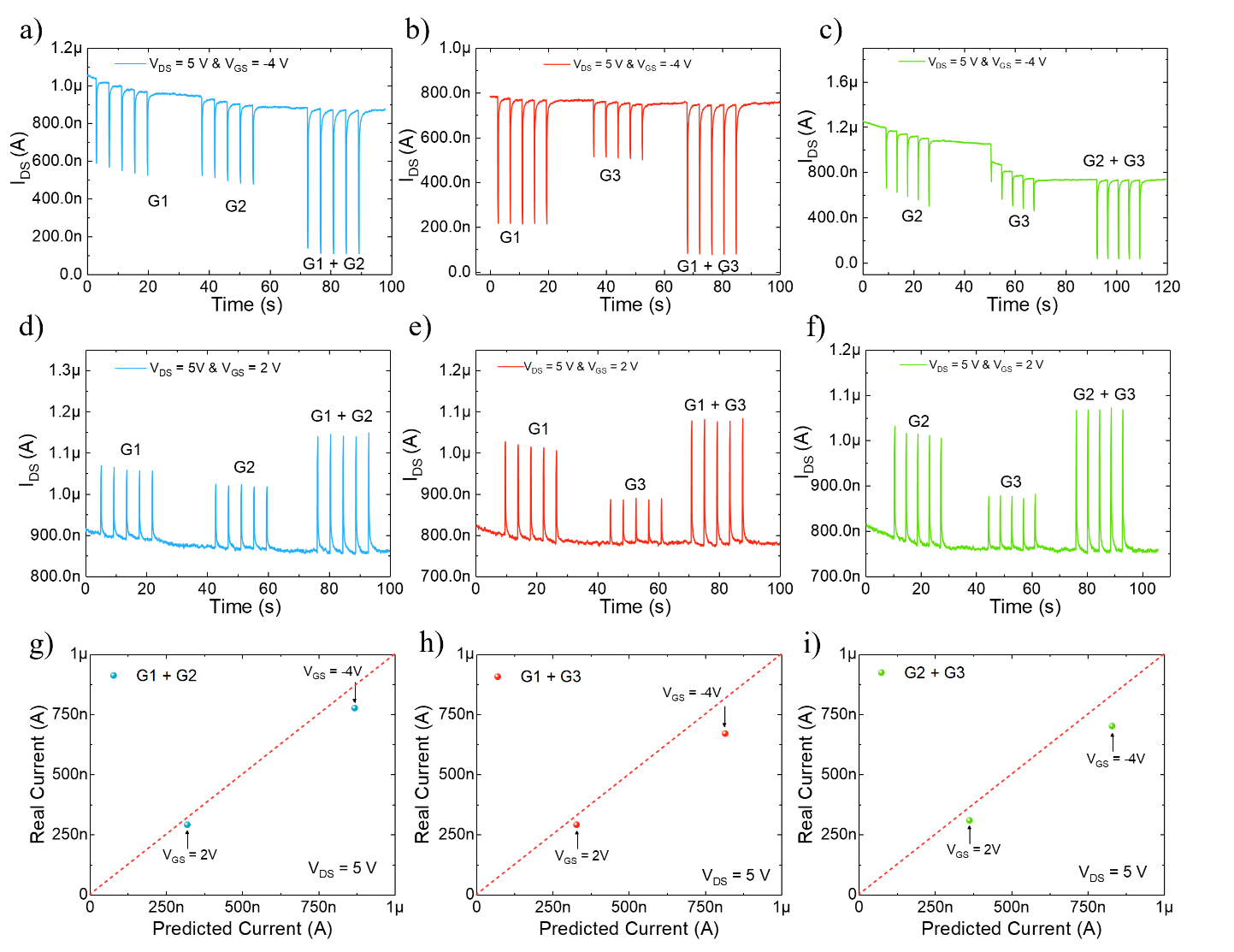


**Figure S5** – Gate summations tests performed by applying V_DS_ = 5V.


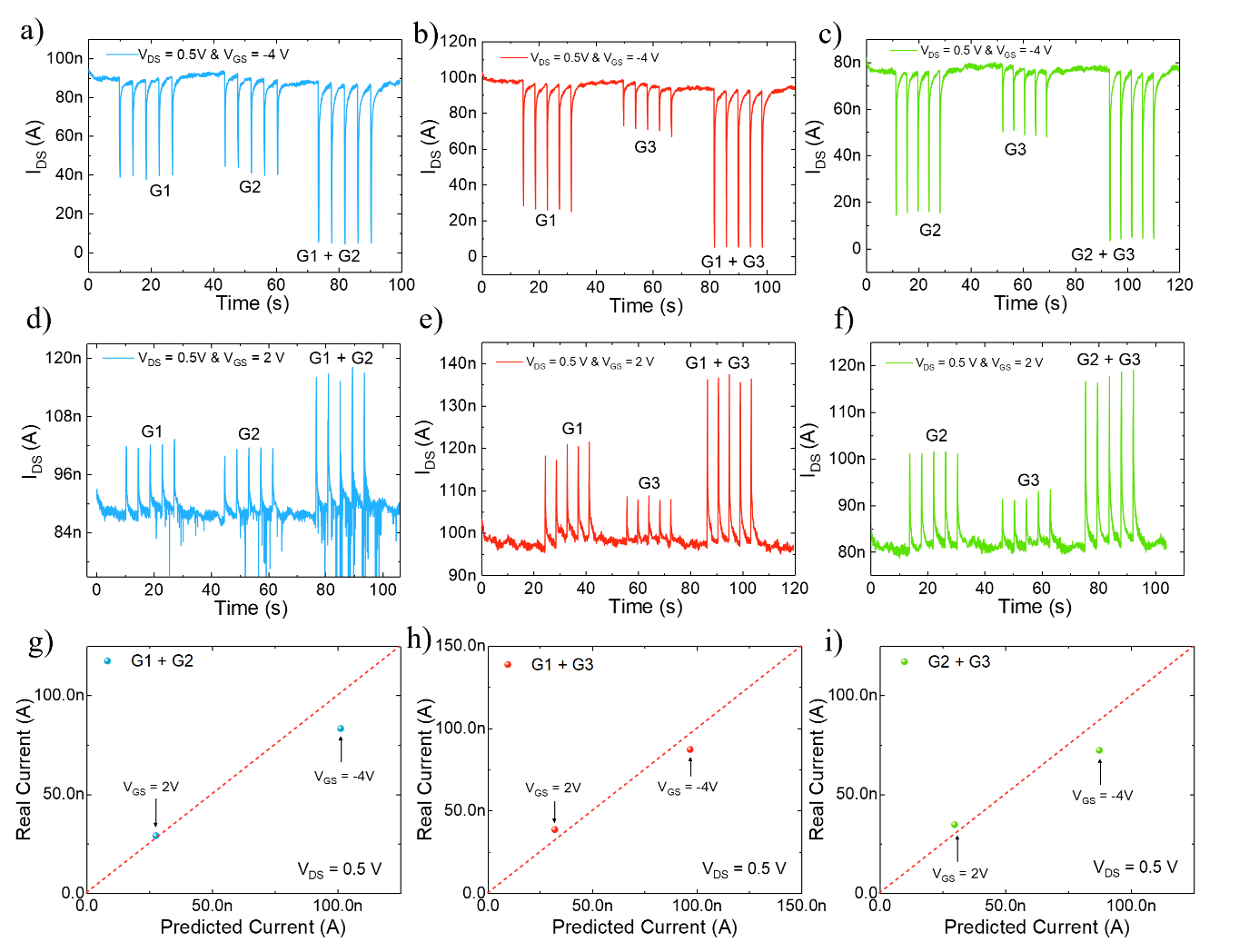


**Figure S6** – Gate summations tests performed by applying V_DS_ = 0.5V


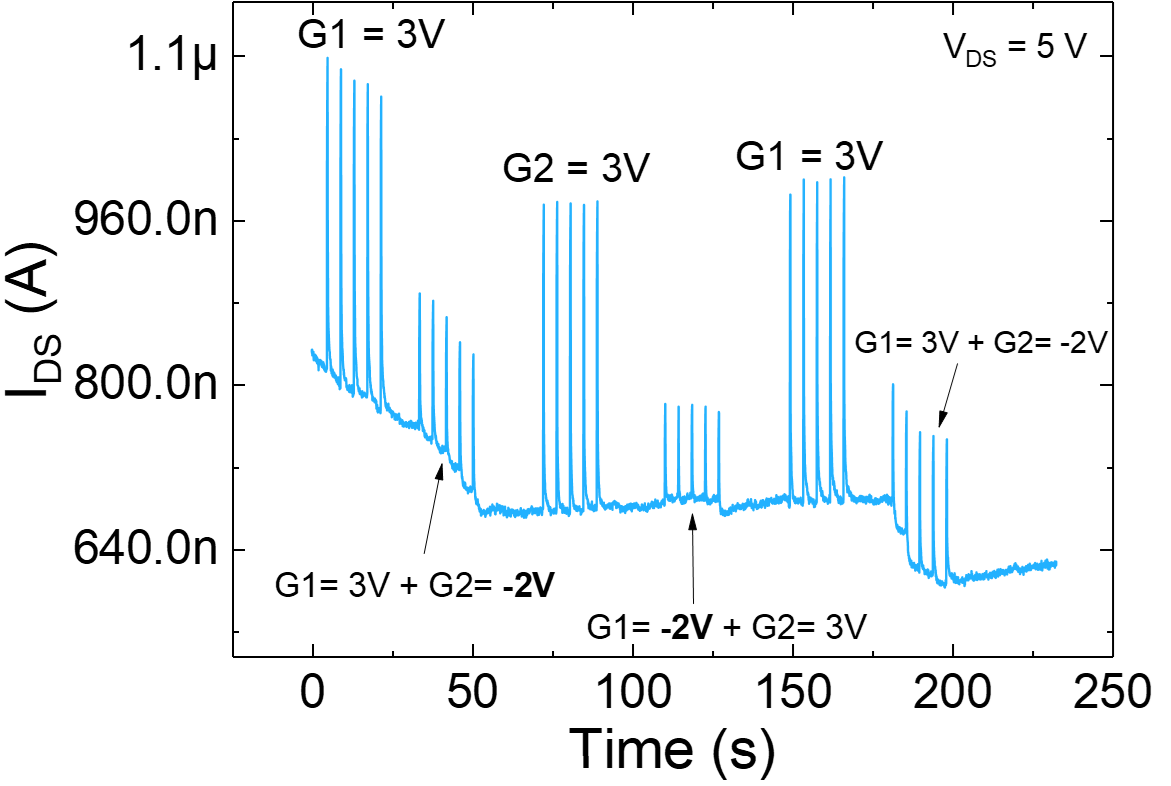


**Figure S7** – Subtraction (inhibitions) tests performed on G1 and G2 with V_DS_ = 5 V.

**References**

[1] H. Morgan, N. G. Green, *Journal of Electrostatics* **1997**, 42, 279.

[2] Y. Liu, J.-H. Chung, W. K. Liu, R. S. Ruoff, *J. Phys. Chem. B* **2006**, 110, 14098.

[3] Y. Yang, W. Guo, X. Wang, Z. Wang, J. Qi, Y. Zhang, *Nano Letters* **2012**, 12, 1919.

[4] M. Liu, W. Su, X. Qin, K. Cheng, W. Ding, L. Ma, Z. Cui, J. Chen, J. Rao, H. Ouyang, T. Sun, *Micromachines (Basel)* **2021**, 12.
